# Comprehensive evaluation of the antibacterial and antibiofilm activities of NiTi orthodontic wires coated with silver nanoparticles and nanocomposites: an in vitro study

**DOI:** 10.1186/s12903-024-05104-w

**Published:** 2024-11-05

**Authors:** Omnia M. Abdallah, Youssef Sedky, Heba R. Shebl

**Affiliations:** 1 Microbiology Department, Faculty of Dentistry, Misr International University, Cairo, Egypt; 2 Orthodontic Department, Faculty of Dentistry, Misr International University, Cairo, Egypt

**Keywords:** Coated orthodontic wires, Antibacterial, Antibiofilm, Silver nanoparticles, Nanocomposite

## Abstract

**Background:**

Fixed orthodontic appliances act as a niche for microbial growth and colonization. Coating orthodontic wires with antimicrobial silver nanoparticles (AgNPs) and nanocomposite was adopted in this study to augment the biological activity of these wires by increasing their antibacterial and antibiofilm properties and inhibiting bacterial infections that cause white spot lesions and lead to periodontal disease.

**Methods:**

Three concentrations of biologically synthesized AgNPs were used for coating NiTi wires. The shape, size, and charge of the AgNPs were determined. Six groups of 0.016 × 0.022-inch NiTi orthodontic wires, each with six wires, were used; and coated with AgNPs and nanocomposites. The antimicrobial and antibiofilm activities of these coated wires were tested against normal flora and multidrug-resistant bacteria (Gram-positive and Gram-negative bacterial isolates). The surface topography, roughness, elemental percentile, and ion release were characterized.

**Results:**

AgNPs and nanocomposite coated NiTi wires showed significant antimicrobial and antibiofilm activities. The chitosan-silver nanocomposite (CS-Ag) coated wires had the greatest bacterial growth inhibition against both Gram-positive and Gram-negative bacteria. The surface roughness of the coated wires was significantly reduced, impacting the surface topography and with recorded low Ni and Ag ion release rates.

**Conclusions:**

NiTi orthodontic wires coated with AgNPs, and nanocomposites have shown increased antimicrobial and antibiofilm activities, with decreased surface roughness, friction resistance and limited- metal ion release.

**Supplementary Information:**

The online version contains supplementary material available at 10.1186/s12903-024-05104-w.

## Introduction

The oral microbiota contains cariogenic acid-producing bacteria that adhere to the tooth surface via biofilm formation and cause demineralization of tooth enamel. Orthodontic arch-wires and brackets placed under the influence of the oral cavity environment which includes the presence of saliva, ingested food, and temperature, in addition to friction or biocorrosion processes. Studies have explained the effect of fixed orthodontic appliances, where they act as plaque-retentive niches by creating stagnant areas with irregular surfaces for brackets, bands, and wires that cause limitations to the cleaning process of the oral cavity; leading to the accumulation of bacterial plaque and aciduric bacterial infections, triggering cavitated lesion periodontal diseases, and the formation of white spot lesions (WSLs) [1]. Studies have shown that 50% of orthodontic patients develop WSL within four weeks or less during their course of treatment [2]. Furthermore, plaque accumulation around orthodontic bands is a slippery slope toward periodontal diseases, where decalcification occurs, followed by the formation of WSLs and gingival inflammation at high precedents reaching 34.4% and 56.8% increases in gingivitis in adult and adolescent patients, respectively [3–6].

During the course of dental treatment, patients are under various types of influences. Specially from Non-oral bacteria that may have access to the oral cavity and take residence in oral cavity and on dental appliances [7]. These microorganisms may be opportunistic pathogens in healthy individual without apparent infections, but can contribute in the drug resistant biofilm formation [7–9]. Furthermore, some bacterial isolates can be acquired during the course of treatment and dental assessment [10].

To prevent the development of various infections, lesions and their consequential diseases, various methods ranging from modifying dietary habits; to applying topical fluoride, forming fluorapatite crystals, and stimulating remineralization and maintaining oral hygiene, have been employed. However, these methods are not effective, and other alternatives have been investigated [4].

The coating of orthodontic appliances can modify the material surface improving the mechanical and biological properties of metallic materials utilized in orthodontics [11]. Different coating methods involving the application of a thin film coating via various techniques, have been proposed. These layers applied to orthodontic wires; may be helpful in enhancing the surface properties of these wires. Coating orthodontic wires will affect their surface roughness, thickness, frictional properties, and ultimately, bacterial adhesion ability. Other methods used fluoride (F)-releasing adhesives and fluoride-releasing elastomeric ligatures. They initially reported that the release of fluoride ions can reduce biofilm formation and control dental caries development [12]. However, further studies showed that fluoridated elastomers were ineffective for long-term use, and their release rate vanished after one week of treatment. Therefore, the need for orthodontic appliances with antibacterial properties is inevitable to lower bacterial adhesion to orthodontic appliances [13].

Nanotechnological solutions were proposed, by providing materials with antibacterial and anti-caries properties that can be used on dental appliances. Nanomaterials, such as silver compounds and nanoparticle (NP) metals, can cause a significant decrease in biofilm formation and therefore inhibit enamel demineralization by acid-producing bacteria [2].

Nickel–titanium (NiTi) arch-wires are an ideal orthodontic appliance for the early stage of comprehensive orthodontic treatment because they generate a light force for dental alignment and levelling. They combine excellent properties, such as a super elastic state with a shape memory effect, making them suitable for biomedical applications. The surfaces of these materials are rough due to their high friction coefficient, resulting in elevated frictional resistance and greater orthodontic forces are needed to overcome this sliding resistance. The surface roughness (SR) of arch-wires is highly important because it determines the surface area in contact and influences frictional, corrosion, and biocompatibility properties [14]. However, the use of NiTi wires is often limited by unstable long-term use; due to erosion by saliva and the release of Ni ions into the oral cavity which can cause health problems [15].

The outstanding properties were determined from the coating materials that have been used, specifically, nanoparticles, where inorganic fullerene-like tungsten disulfide (WS_2_), zinc oxide (ZnO), chitosan (CS) and carbone Nitride (CNx) nanoparticles significantly decrease the friction coefficient [16–18].

Silver nanoparticles (AgNPs) are among the most promising antimicrobial agents with a wide range of applications such as wound and burn healing, as well as in bone and dental implants, benefiting from their antibacterial activities [19]. The synthesis of AgNPs using bacterial isolates as biofactors, which mediate the nucleation and growth processes of AgNPs provided an effective metabolic pathway for the bio-formation of nanoparticles with well-defined shapes, high reactivity, and water solubility properties. These findings qualify biogenically, i.e., using bacteria as bio-factory and eco-friendly synthesized AgNPs as the favored choice for various biomedical applications. This route is more favored than physical or chemical methods because substantial amounts of energy and toxic solvents used for nanoparticles extraction, which limits its use in biomedical applications. The biogenic pathway provides nanoparticles with a controlled shape, low aggregation rates, high homogeneity, with a low polydispersity index (PI), and high stability [20, 21].

These AgNPs have broad antibacterial activity against Gram-positive and Gram-negative bacteria. Beside the well-known oral bacterial inhabitants such as *Staphylococcus spp*, *Streptococcus spp* and *Enterococcus spp*, other microbial non oral pathogens have used the oral cavity as a reservoir [7]. Coating dental appliances with nanoparticles such as AgNPs is critical part of recent research and focusing on evaluating this new advancement against drug resistant bacterial isolates and aggressive biofilm forming bacterial such as *Acinetobacter baumannii and Pseudomonas aeruginosa.* Other studies have stated these microbial infections in the oral cavity as code blue alert, to be focused on in dental research, as the emergence of non-oral pathogens is gaining a lot of research interest due to their effect and refractory to endodontic and periodontal treatments [8, 22, 23]. Nanostructured silver particles are highly accessible to bacterial cells due to their nano-size range and high surface-to-volume ratio, leading to elevated efficacy in anchoring to the microorganisms’ cell structure and penetrating the cell membrane, forming free radicals, damaging DNA, causing structural changes, and ultimately causing cell death. AgNPs can release Ag ions from silver clusters at continuous rates, ensuring antimicrobial durability [24].

These nanoparticles can bind to polymers to form nanocomposites that gain antimicrobial and antibiofilm properties from the encapsulated particles. Polyvinyl alcohol (PVA) and chitosan (CS) have been integrated in other applications as they are biodegradable and biocompatible. PVA is FDA approved for use in the food industry and medical applications without side effects. CS is a natural biodegradable, nontoxic polymer that can help in buffering the acidic oral environment and reducing tooth demineralization. Both PVA and CS can be used as biological scaffold for nanoparticles, leading to a wider range of applications [25].

Here, NiTi orthodontic wires were separately coated with AgNPs and nanocomposites, using the dip coating method. This procedure is part of sol-gel process that is best known for their stoichiometry stability, purity, and ensures homogeneity, which makes it suitable for coating materials at low cost by only changing the solution composition. Furthermore, it aims to ensure the binding and stability of the synthesized nanoparticles and nanocomposites on the tested surface [11, 26, 27].

This study aimed to evaluate the antimicrobial and antibiofilm efficacy of coating NiTi orthodontic wires with five materials, CS, PVA, AgNPs, CS-Ag, and PVA-Ag, on wire surfaces using the sol-gel thin-film dip coating method. The effects of these coated and uncoated control wires on multidrug-resistant and oral flora bacteria were tested. Furthermore, the topography and surface roughness fluctuation of these coated vs. uncoated wires were assessed and the release of silver and nickel ions from these wires was calculated.

## Methods

**Fig. 1:**
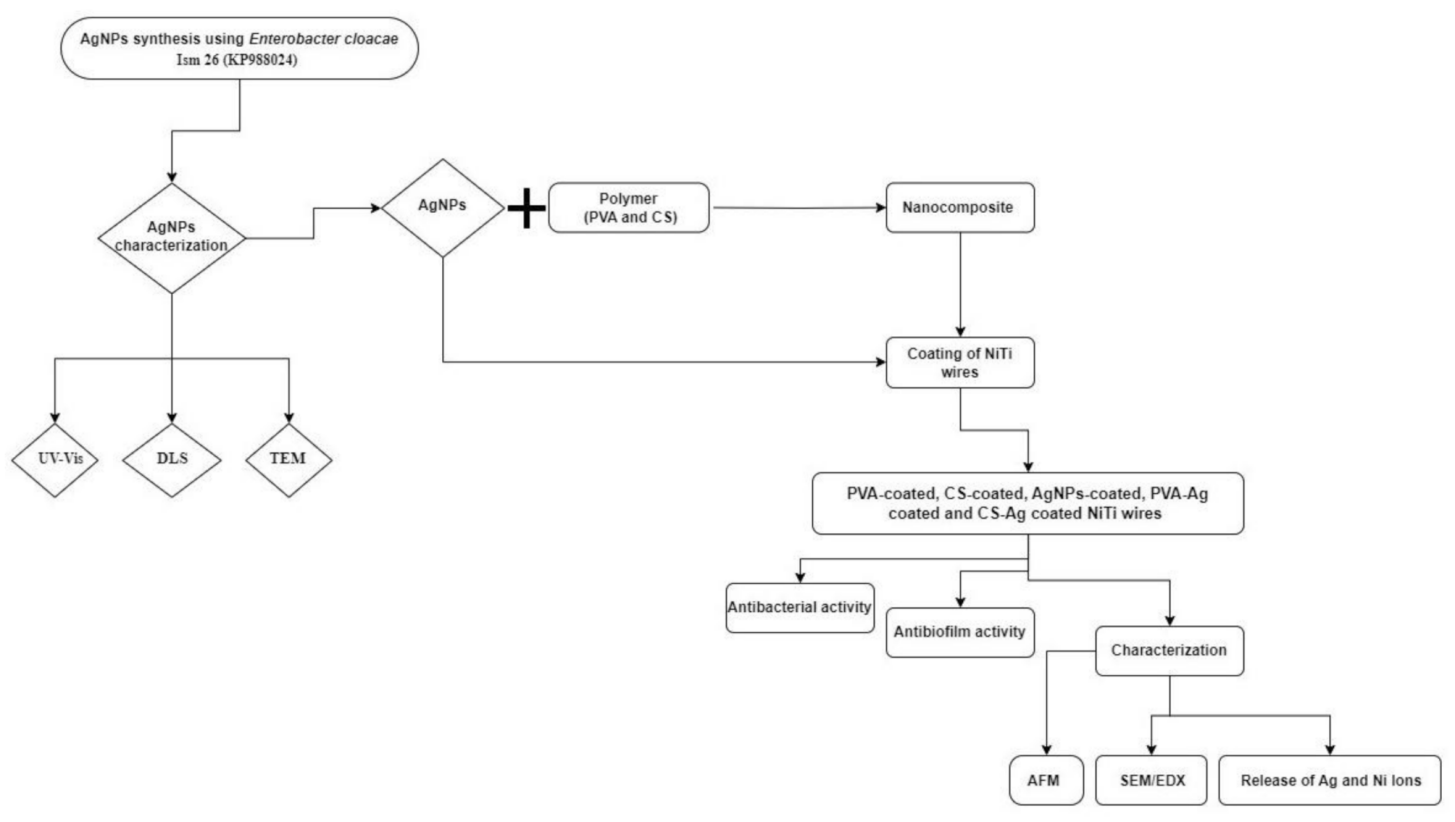
Flowchart diagram that presents the steps involved in the study to synthesize AgNPs, Nanocomposites, and NiTi wires coating

The use of biologically synthesized AgNPs and Ag nanocomposite as a coating layer on the surface of NiTi wires was evaluated and depicted in a flowchart showing the steps of this study (Fig. 1). This power analysis used the adhesion of bacteria to the wire as the primary outcome. Based on the results of Gonçalves, et al. 2020 [28] the mean and standard deviation (SD) values were 0.174 (0.04) and 0.118 (0.007) for the uncoated and coated wires, respectively. The effect size (d) was 1.95. Using an alpha (α) level of 5% and beta (β) level of (20%) i.e. power = 80%; the minimum estimated sample size was 6 wires per group. Sample size calculation was performed using G*Power Version 3.1.9.2.

The wires were divided into six groups, each with six wires: an uncoated control group (wires with no treatment), a CS coated group, a PVA coated group, a AgNPs coated group, a CS-Ag nanocomposite-coated group, and a PVA-Ag nanocomposite-coated group.

### Synthesis of AgNPs and nanocomposites

Biologically synthesized AgNPs were prepared in our laboratory using *Enterobacter cloacae* Ism 26 (KP988024) [29]. Briefly, 100 mL of nutrient broth medium was inoculated with 100 µL (10^8^ CFU) of KP988024 and incubated at 35 °C and 180 rpm for 24 h. The bacterial culture was then centrifuged at 7000 rpm for 10 min. The bacterial pellets were collected, washed, and sonicated after which the bacterial cell lysate supernatant was mixed with 1 mM AgNO_3_ solution and incubated at 35 °C for 24 h. the synthesized AgNPs solution was obtained in powder form by lyophilization using an Edwards model RV5 (England). Different solutions were prepared at three concentrations (0.1% (C1), 0.2% (C2) and 0.3% (C3) (w/v)) in deionized sterilized water and used for further experiments. Briefly, for the synthesis of PVA-Ag and CS-Ag nanocomposites, PVA (alpha cheimeka, India) (2 g) was added to 18 ml of deionized water and magnetically stirred at 100 rev/ min on a hot plate at 90 °C for 3 h. Then, different concentrations of the biosynthesized AgNPs were added and stirred for another 4 h (0.1% (C1), 0.2% (C2) and 0.3% (C3) (w/v)). Chitosan (alpha cheimeka, India) (0.4 g) was added to 20 ml of acetic acid (1%) and magnetically stirred for 1 h at 60 °C; then, 0.1%, 0.2% and 0.3% (w/v) AgNPs were added, and the mixture was further stirred for 2 h. These nanocomposite solutions were used for further experiments and pure PVA and CS solutions were used as controls [30].

### Characterization of biogenically synthesized AgNPs

#### UV-Vis spectroscopy

Analysis was carried out to assess the presence of AgNPs by using 1 mL of brown-colored solution sample in a quartz cell where the presence of the specific peaks (400–450 nm) for AgNPs was assessed using an Evolution 201 Scan UV-visible spectrophotometer (Thermo Scientific). Measurements were performed between 200 and 800 nm in the range of 1 nm. To set up the baseline we used double distilled water as a blank.

#### Dynamic light scattering (DLS)

The particle size and charge of the AgNPs in the solution were measured by DLS using a PSS-NICOMP particle sizer 380ZLS and zeta sizer (Malvern Instruments Ltd.).

#### Transmission electron microscopy (TEM)

TEM was used to accurately determine the shape and size in nm of the synthesized AgNPs. JOEL JEM-1010, Transmission electron microscopy at 80 KV was used at the Regional Centre for Mycology and Biotechnology (RCMB) of Al-Azhar University.

### Coating NiTi wires with AgNPs and ag nanocomposites

Here, we used the sol-gel thin film dip coating method, where round 0.016 × 0.022-inch orthodontic NiTi wires (OrthoPro, FL, USA) were cleaned using an ethyl alcohol solution. NiTi wires were dip-coated in the previously prepared solutions with different AgNPs concentrations (AgNPs, PVA-Ag, CS-Ag) and pure solutions of PVA and CS and left for 8 h. Then all the samples were grasped with sterilized tweezers and left to air dry in a laminar flow hood overnight. Finally, the coated wires were placed in an oven at 40 °C for 10 min as a final step before use in further experiments. Uncoated wires were used as control. All procedure steps were conducted in a laminar flow, and all samples were kept separate and sealed in sterilized Eppendorf tubes until the next experiment [6, 16].

### Antibacterial activity

The antibacterial activity of all the coated and uncoated samples was evaluated using an agar diffusion test according to the Clinical and Laboratory Standards Institute (CLSI). Mueller-Hinton agar (MHA) (Oxoid Ltd., England) was sterilized and inoculated with the bacterial culture. This test was performed against clinically relevant Gram-positive and Gram-negative bacterial microorganisms commonly used as standards (three multidrug-resistant bacteria, *Acinetobacter baumannii* (A1), *Acinetobacter baumannii* (A2), and *Pseudomonas aeruginosa* (P1)), and three other common bacterial oral flora, *Staphylococcus aureus* (S1), *Streptococcus mutans (*ATCC 25175) (St1), and *Enterococcus faecalis* (ATCC 19433) (E1) ). These bacterial strains were obtained from Ain Shams University Hospital, Cairo, Egypt; these strains are endemic to society and present as oral microbial flora [31].

MHA plates were inoculated with 10^6^ CFU/ml bacterial cultures using a sterile cotton swab, and each tested bacterial isolate was uniformly spread on culture media, separately and under aseptic conditions. Six orthodontic wires were placed on each plate (uncoated control, PVA-coated, CS-coated, AgNPs-coated, PVA-Ag coated, and CS-Ag coated) then incubated at 37 °C for 24 h. The antimicrobial assay was performed in triplicate, and the diameter of the inhibition zone (mm) was measured; the results are reported as the mean ± standard deviation [13, 30].

### Antibiofilm activity

The ability of the coated wires to inhibit biofilm formation was evaluated against two strong biofilm-forming bacteria (A1 and P1). Briefly, bacterial isolates were grown in sterile (10 ml) tryptic soy broth (TSB) (Merck, Germany). Bacterial inoculum of 0.5 McFarland standard (1.5 × 10^8^ CFU/ml ) was used and diluted by 1:100, then inoculated into 96-well microtiter plates that previously contained coated and uncoated wires. The plates were incubated at 37 °C for 24 h and then washed and air dried at room temperature. Crystal violet solution (200 µl of 0.1% w/v) was added, and the mixture was left for 30 min and then washed and dried. Finally, 100 µl of ethanol (96%) was added to extract the stained bound biofilm. The absorbance was measured at 490 nm by microplate reader (ELx808™ Absorbance, Biotek, USA). Biofilm reduction was measured and compared to that of the control and the percentage of biofilm inhibition was calculated using the following equation: [30, 32]

$$\displaylines{\% ofinhibition = \cr 1 - \left( {{{ODofcoated\,wires} \mathord{\left/{\vphantom {{ODofcoated\,wires} {OD\,ofnegativecontrol}}} \right.\kern-\nulldelimiterspace} {OD\,ofnegativecontrol}}} \right) \times 100 \cr}$$

The OD of the tested wire is the optical density of the sample.

The OD of the negative control is the optical density of the control biofilm-forming bacteria.

### Characterization of NiTi coated wires

#### Atomic force microscopy (AFM)

The surface characteristics of the coated wires were assessed by AFM (NanoSurf C3000, Gräubernstrasse, Liestal, Switzerland) operating in phase contrast mode. AFM provides measurements of surface roughness, and variability in defined measured areas by providing 3D images. The average coated layer thickness, roughness (Ra), and maximum roughness depth (Rq) were calculated for the coated wires using image processing and data analysis software supplied with the AFM.

#### Scanning electron microscopy (SEM)/ energy dispersive X-ray spectroscopy (EDX)

Wire specimens were examined by SEM/EDX (QUANTA, FEG 250, Thermo Scientific) operating at an accelerating voltage of 20 kV to visualize the surface changes and detect the coating extent and its efficacy on wires for all tested coating materials vs. uncoated control wire samples EDX analysis was used to identify the composition and elemental analysis the specimen surfaces. The wire specimens were mounted on metallic copper stubs and fixed with carbon conductive tape at a standard tilt angle, and SEM photomicrographs were taken from the surface at various magnifications [4, 28].

#### Release of Ag and Ni ions

Nickel and Ag ion release analysis was performed by immersing the NiTi arch-wires (uncoated and coated) in 5 mL of phosphate-buffered saline (PBS) in a sterilized tube for a period of 192 h. The released Ag and Ni ions were measured using Atomic Absorption Spectrometer (AAS) (Perkin Elmer 3100) at 24, 48, 96 and 192 h for each wire sample.

### Statistical analysis

The SPSS standard software package was used for data analysis. One-way analysis of variance (ANOVA) with Tukey’s post-hoc test was used to compare the effects between the groups (*n* = 6) for antibacterial and antibiofilm tests. The data are represented as the mean ± standard deviation (SD). The level of significant difference was set at *p* < 0.05.

## Results

### Characterization of AgNPs

**Fig. 2:**
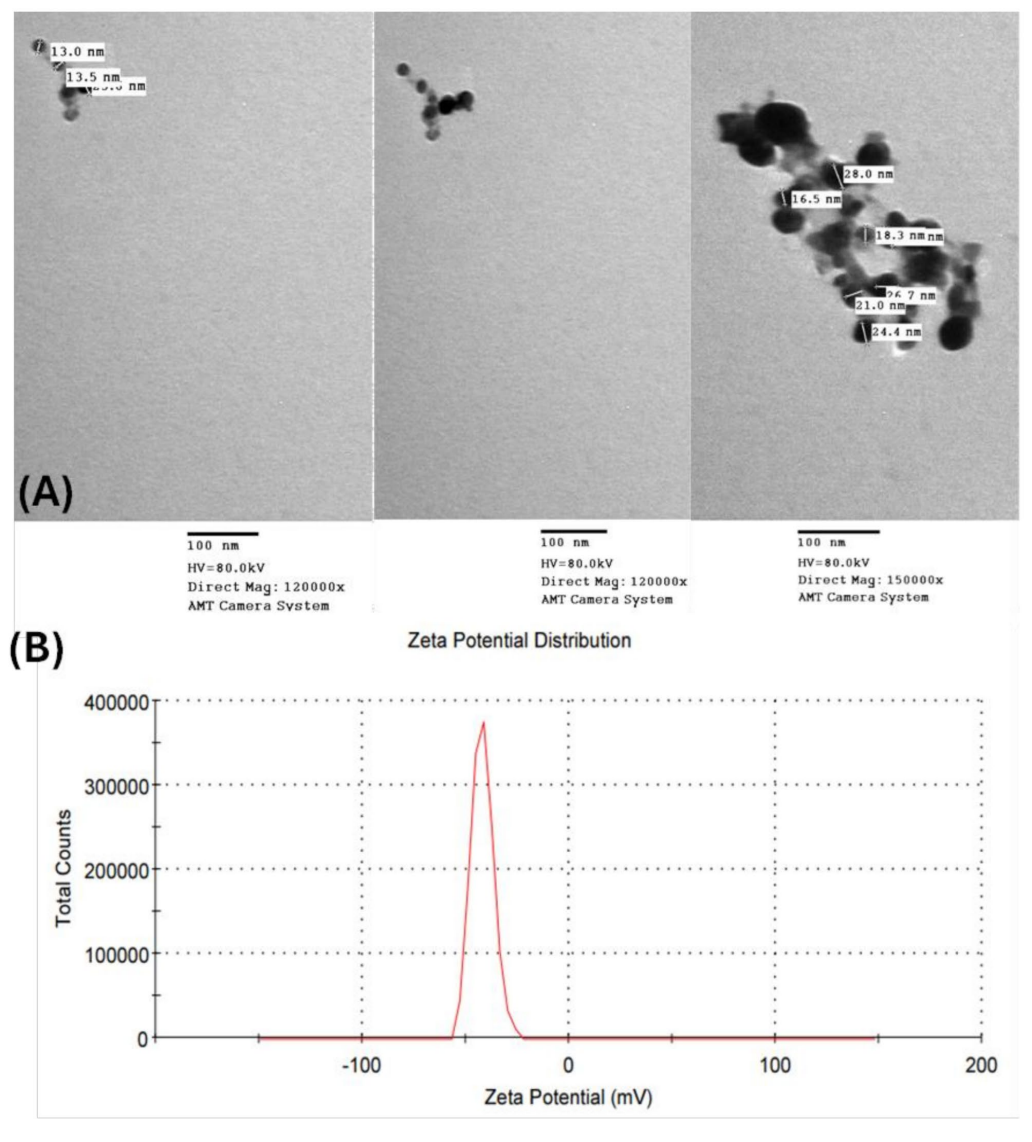
Characterization of AgNPs biosynthesized from *Enterobacter cloacae* Ism 26 (KP988024). (**A**) TEM micrographs of AgNPs. (**B**) Zeta potential analysis

*Enterobacter cloacae* Ism 26 (KP988024) was able to synthesize AgNPs by forming a reddish-brown solution which further showed characteristic peak of the AgNPs at 430 nm. TEM micrographs showed nanoparticle sizes ranging from 30 to 13 nm. The AgNPs were spherical in shape, with an average size of 20 nm and negatively charged, with zeta potential of (-42 mV) (Fig. 2) [29].

### Antibacterial activity

The antimicrobial activity of the coated and uncoated NiTi wires (uncoated control, PVA-coated, CS-coated, AgNPs-coated, PVA-Ag coated, and CS-Ag coated) at different concentrations of AgNPs (C1, C2 and C3) was assessed against MDR bacteria using an agar diffusion assay.

**Fig. 3:**
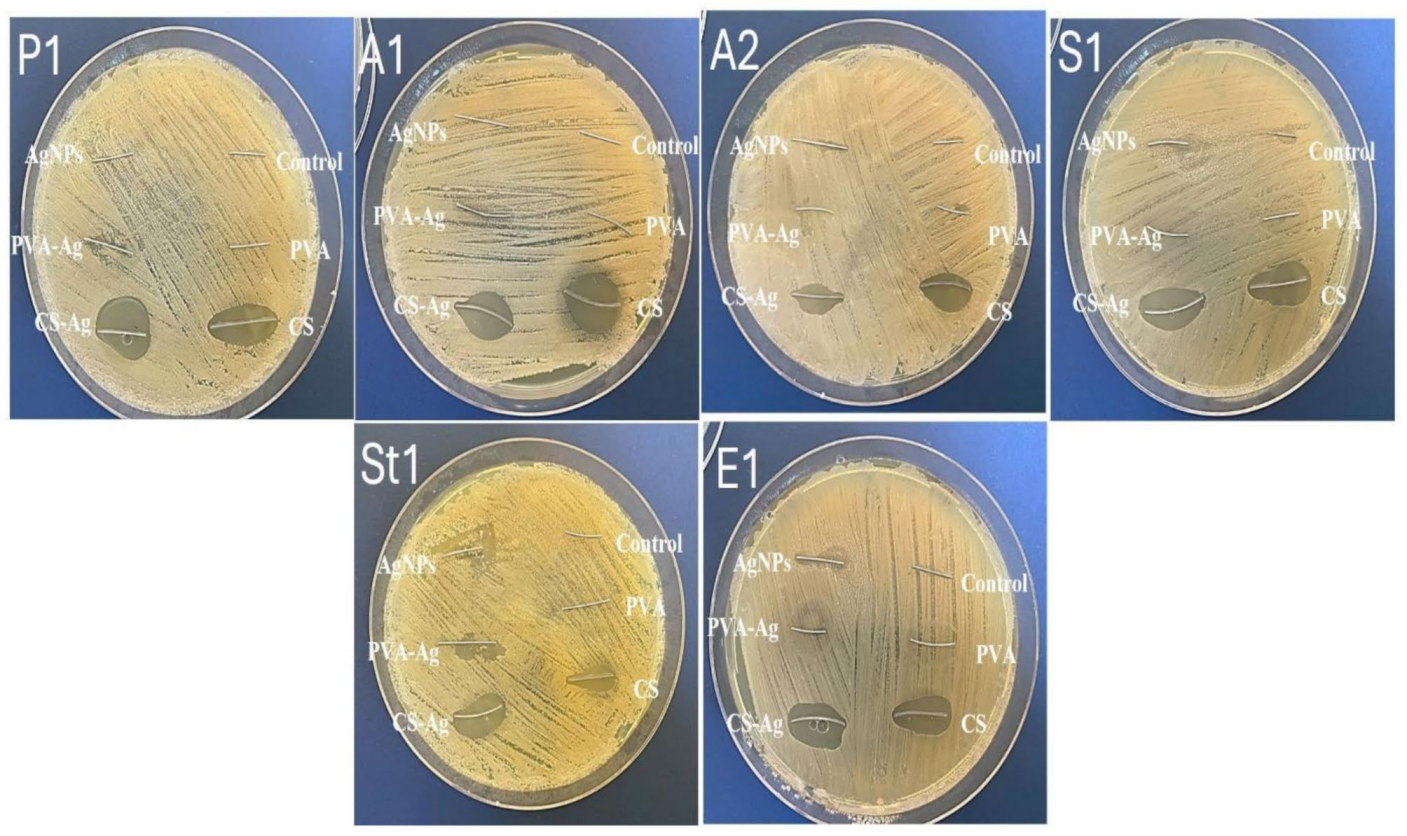
Antibacterial activity of control uncoated wires vs. coated wires using an agar diffusion assay P1, *Pseudomonas aeruginosa*; A1, *Acinetobacter baumannii*; A2, *Acinetobacter baumannii*; S1, *Staphylococcus aureus*; St1, *Streptococcus mutans*; and E1, *Enterococcus faecalis*

CS-Ag coated NiTi wires showed the most significant antimicrobial activity at different concentrations, where C3 showed the largest inhibition zones, with diameters ranging between 20.33 ± 0.816 and 26.16 ± 1.329 mm, against both Gram-positive and Gram-negative bacteria, followed by PVA-Ag coated at C3, with inhibition zones ranging from 18.5 ± 0.816 to 25.33 ± 0.516 mm. Both PVA-Ag coated, and CS-Ag coated showed the maximum activity against S1, and the minimum activity against P1 and St1. Furthermore, the relationship between the concentration of AgNPs and the inhibition zone was directly proportional for both Gram-positive and Gram-negative bacteria. The uncoated control and PVA-coated NiTi wires showed zero inhibition zones against all the evaluated bacterial isolates. However, the CS-coated wires showed similar, sometimes even greater inhibition zone values than did the AgNPs coated wires (Fig. 3; Table 1).

**Table 1:** Inhibition zone diameters of coated and uncoated NiTi wires against bacterial isolates

**C1**	**control**	**AgNPs**	**PVA**	**PVA-Ag**	**CS**	**CS-Ag**
A1	0	0	0	13.33 ± 1.63^a^	11.66 ± 1.21 ^b^	15.16 ± 1.47^c^
A2	0	0	0	16.33 ± 1.65 ^a^	14 ± 1.26 ^b^	11 ± 1.26^c^
S1	0	5.66 ± 1.51^a^	0	14.83 ± 1.16 ^c^	10.33 ± 0.81 ^b^	17 ± 1.09^d^
P1	0	0	0	8.83 ± 1.17 ^a^	10 ± 0.63^b^	16 ± 1.27^c^
St1	0	0	0	16.33 ± 1.21 ^a^	13.5 ± 1.04 ^b^	18.5 ± 1.51^c^
E1	0	0	0	16.4 ± 2.06 ^a^	13.16 ± 1.94^b^	8.5 ± 1.04^c^
C2	**control**	**AgNPs**	**PVA**	**PVA-Ag**	**CS**	**CS-Ag**
A1	0	8.16 ± 0.98 ^a^	0	0	11.66 ± 1.21 ^b^	21.66 ± 1.21^c^
A2	0	11.66 ± 1.21^a^	0	0	14 ± 1.26^b^	19.5 ± 0.83^c^
S1	0	7.33 ± 1.36 ^b^	0	9.16 ± 0.98 ^a^	10.33 ± 0.81^c^	15 ± 1.26^d^
P1	0	0	0	0	10 ± 0.81^b^	14.33 ± 0.81^c^
St1	0	0	0	0	13.5 ± 0.98^b^	19.16 ± 0.98^c^
E1	0	0	0	0	13.16 ± 1.94^b^	15.16 ± 0.75^c^
C3	**Control**	**AgNPs**	**PVA**	**PVA-Ag**	**CS**	**CS-Ag**
A1	0	9.5 ± 1.37 ^a^	0	23.83 ± 0.40^c^	11.66 ± 1.21^b^	24.66 ± 0.51 ^c^
A2	0	10.16 ± 2.31 ^a^	0	22.83 ± 0.75^c^	14 ± 1.26^b^	23 ± 0.63^c^
S1	0	20.16 ± 1.16 ^b^	0	29.66 ± 0.51^d^	10.33 ± 0.81^a^	26.16 ± 1.32^c^
P1	0	0	0	18.5 ± 0.81^b^	10 ± 0.63^a^	21.83 ± 0.75^c^
St1	0	16.83 ± 1.16 ^b^	0	25.33 ± 0.51^d^	13.5 ± 1.04^a^	20.33 ± 0.81^c^
E1	0	21.16 ± 0.98 ^c^	0	19.33 ± 1.03^b^	13.16 ± 1.94^a^	23.33 ± 0.81^d^

(C1 (0.1%), C2 (0.2%) and C3 (0.3%) (w/v) of silver nanoparticles), (AgNPs: silver nanoparticles, PVA: polyvinyl alcohol, PVA-Ag: nanocomposite of polyvinyl alcohol and silver nanoparticles, CS: chitosan, CS-Ag: nanocomposite of chitosan and silver nanoparticles. (A1) *Acinetobacter baumannii*, (A2) *Acinetobacter baumannii*, (P1) *Pseudomonas aeruginosa*, (S1) *Staphylococcus aureus*, (St1) *Streptococcus mutans* and (E1) *Enterococcus faecalis.* The results are presented as the means ± SDs (*n* = 6). The data were analysed via one way ANOVA followed by post hoc analysis, and the ^a−d^ superscript means are significantly at p˂0.05; for the uncoated control vs. the coated wires

### Antibiofilm activity

**Fig. 4:**
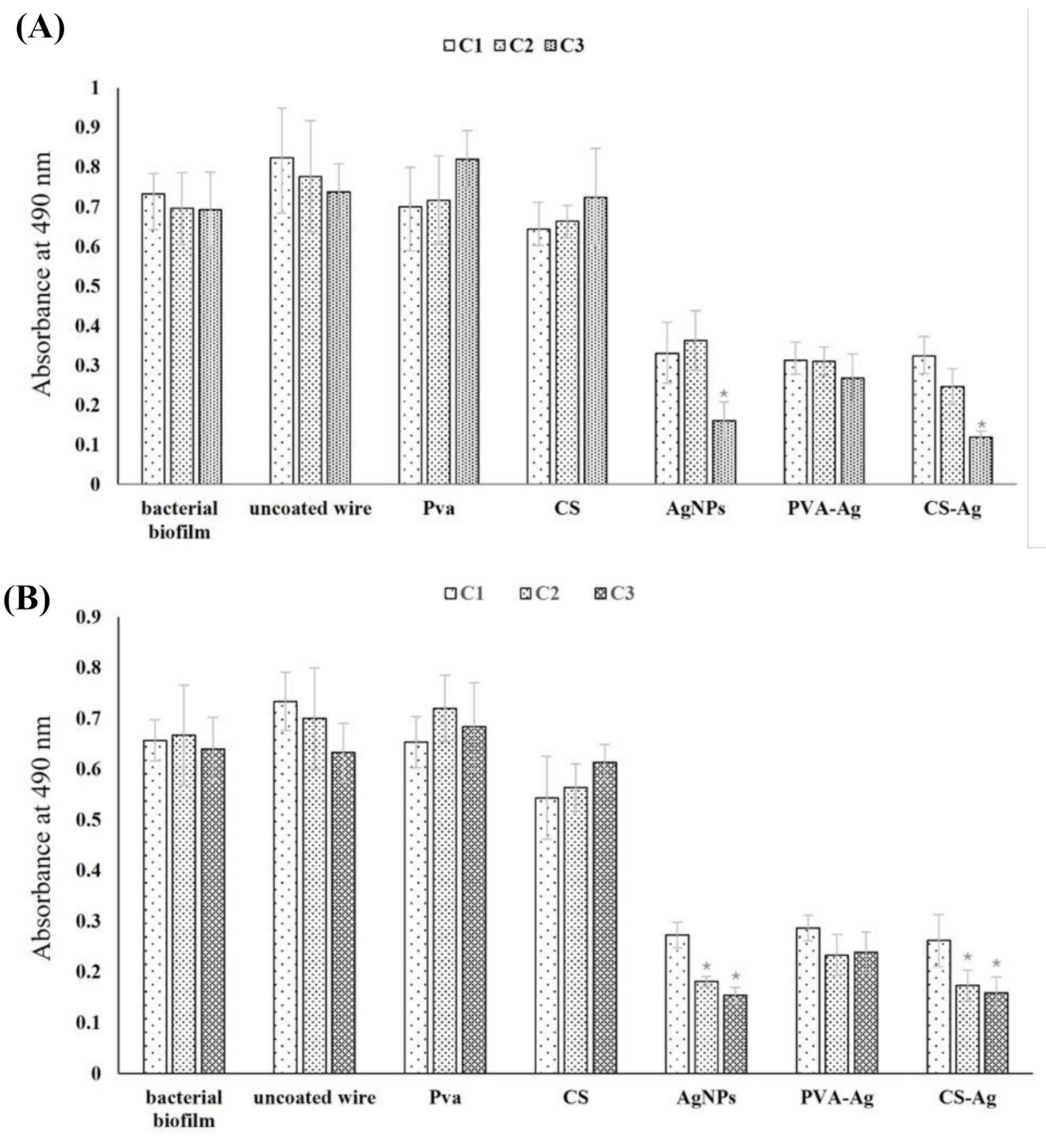
Antibiofilm activity of control uncoated wires vs. coated wires against biofilm-forming bacteria. ((0.1% (C1), 0.2% (C2) and 0.3% (C3) (w/v)of silver nanoparticles). The results are expressed as the mean ± SD (*); P˂0.05. (**A**)*Pseudomonas aeruginosa* (P1), (**B**)*Acinetobacter baumannii* (A1)

Coated and uncoated NiTi wires were evaluated for their antibiofilm activity against biofilm-forming bacterial isolates (A1 and P1). In this test we have calculated the dead and inhibited cells not the metabolic activity of the biofilm. Both bacterial isolates showed high absorbance at 490 nm with biofilm formation against control uncoated NiTi wires; furthermore, this absorption increased with PVA-coated wires, showing the same low range of biofilm inhibition even as the concentration of AgNPs increased. Although the presence of CS as a coating material caused a slight decrease in biofilm formation, augmenting CS with AgNPs to form a nanocomposite coating layer has caused significant biofilm inhibition which was significantly observed as the concentration of AgNPs increased at C3, reaching 83 ± 3.5% inhibition against (A1) *Acinetobacter baumannii* biofilm-forming bacteria (Fig. 4).

### Characterization of the NiTi coated wires

#### Atomic force microscopy (AFM)

**Fig. 5:**
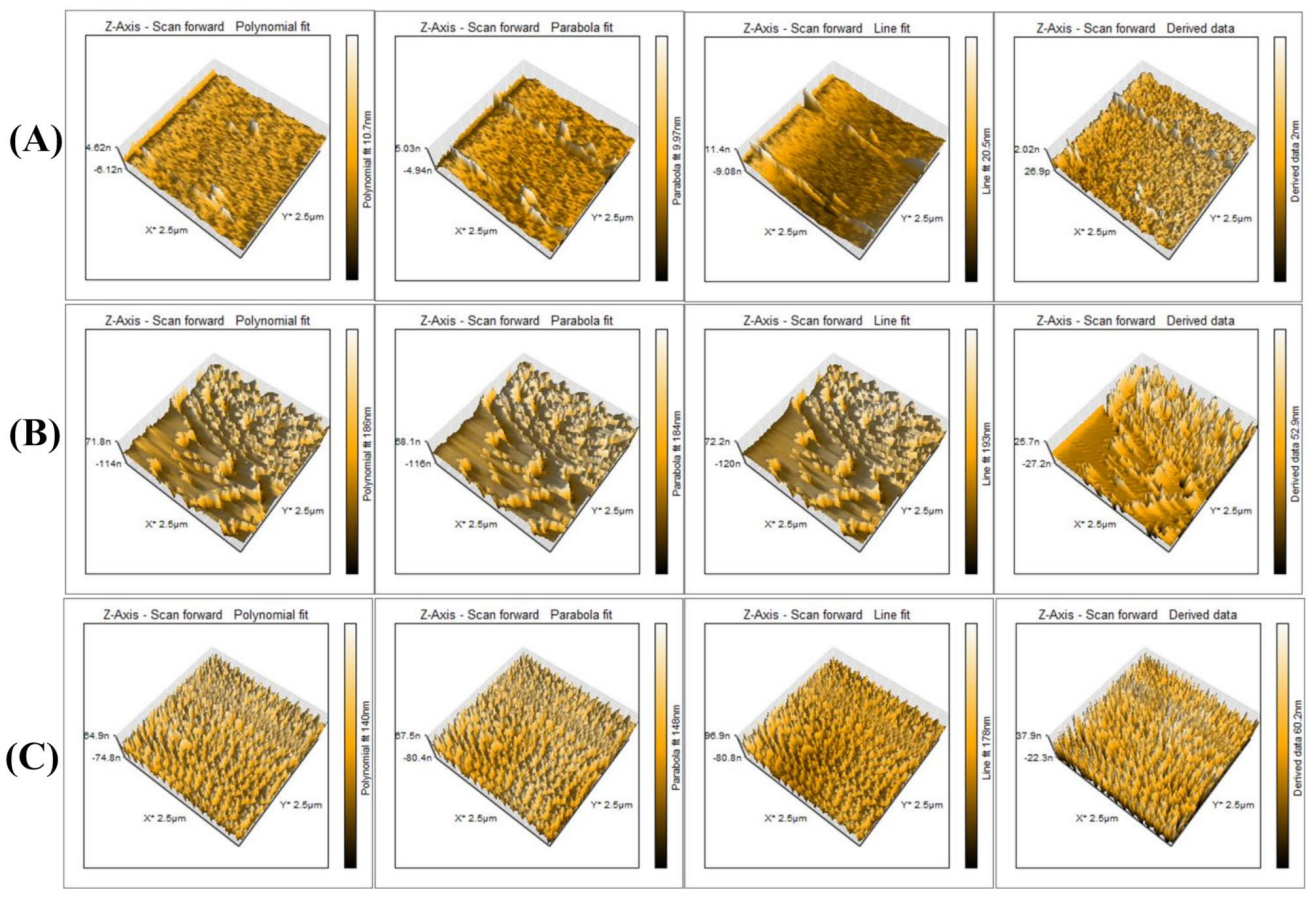
Atomic force microscopy 3D images of NiTi wires coated with three different materials. The scanned area was 2.5 μm×2.5 μm with the peak height of the coating for each material. (**A**) PVA-Ag, (**B**) CS-Ag and (**C**) AgNPs

The AFM 3D image pseudocolor graphs show the different coating material attachments and surface differences between thickness and appearance of the three coated NiTi wires (Fig. 5). The observed coated layers showed variability in thickness, where the PVA-Ag layer showed the lowest thickness at an average of 10 nm, while the AgNPs coating layer showed an intermediate line fit thickness at an average of 178 nm. The CS-Ag coating layer showed the greatest thickness at an average of 193 nm. The average Ra values for the PVA-Ag, CS-Ag, and AgNPs coated NiTi wires were 40.06 nm, 139.99 nm, and 40.33 nm, respectively. Furthermore, the average Rq for the PVA-Ag, CS-Ag, and AgNPs coated wires were 46.3 nm, 164.12 nm, and 49.58 nm, respectively.

#### Scanning electron microscope equipped with energy dispersive X-ray spectroscopy (SEM/EDX)

**Fig. 6:**
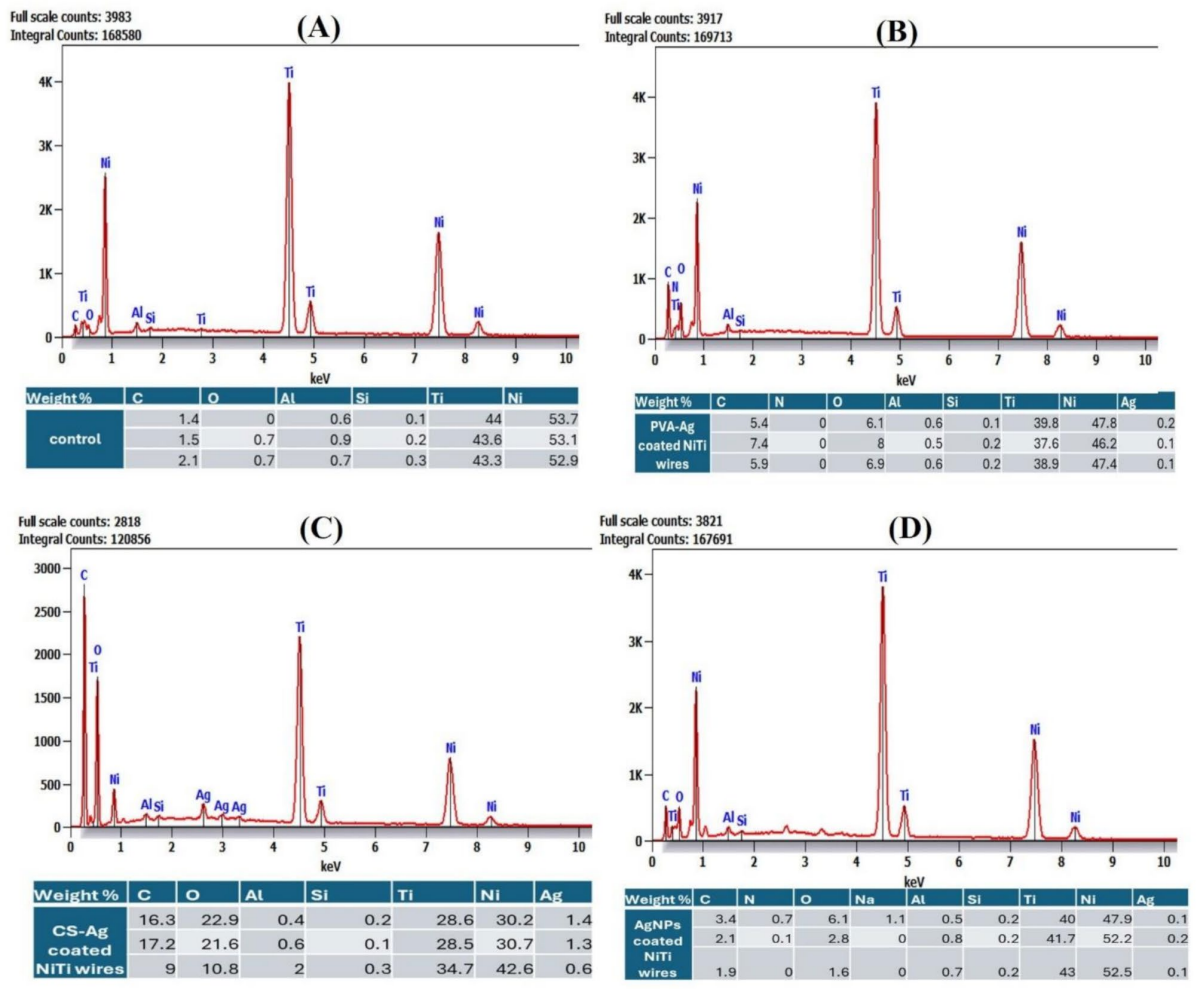
EDX analysis with element percentiles of control uncoated and coated NiTi orthodontic wires. (**A**) Uncoated control wire, (**B**) PVA-Ag coated wires, (**C**) CS-Ag coated wires, and (**D**) AgNPs coated wires

**Fig. 7:**
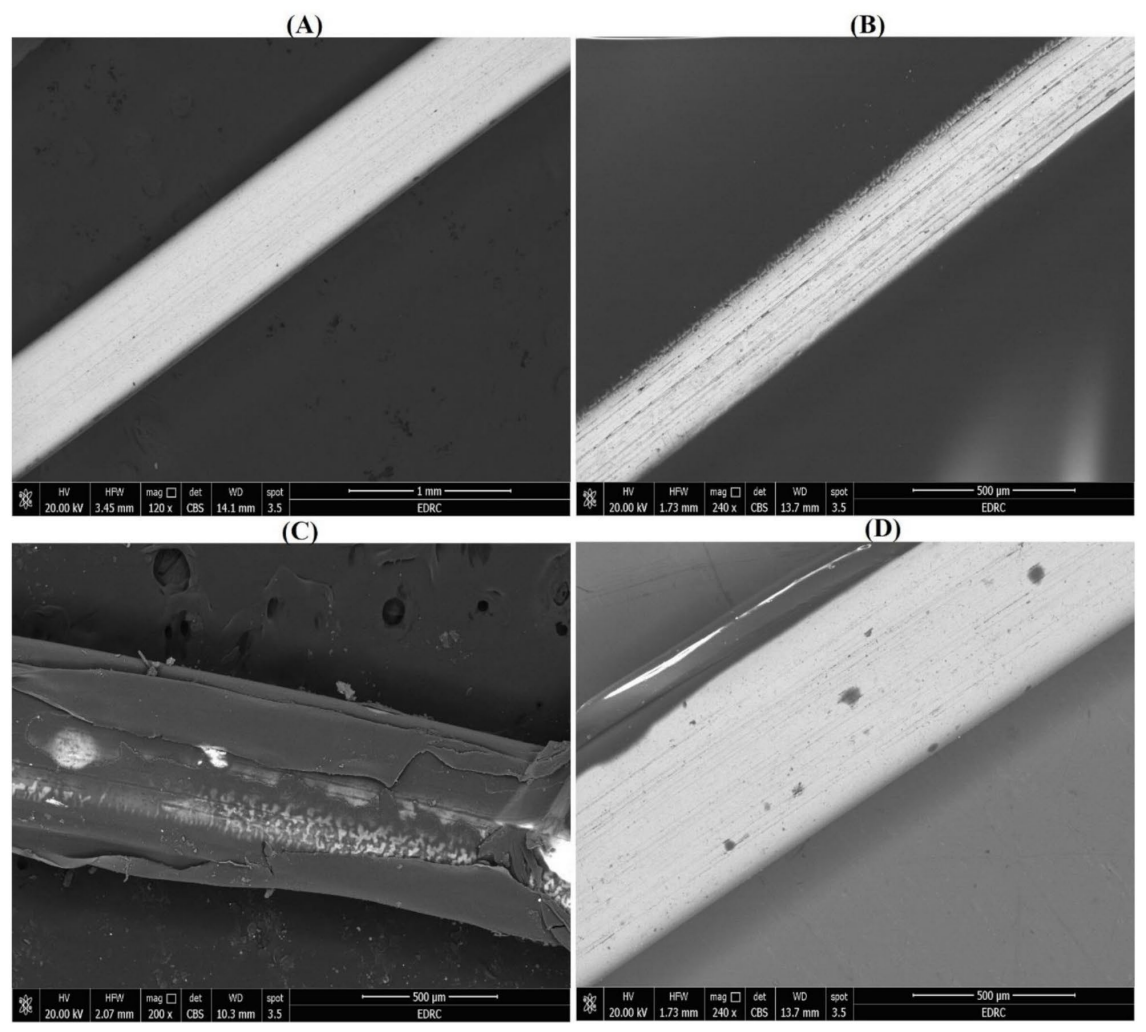
SEM images at different magnifications control uncoated and coated NiTi orthodontic wires. (**A**) Uncoated control wire, (**B**) PVA-Ag coated wire, (**C**) CS-Ag coated wire, and (**D**) AgNPs coated wire

SEM images of the coated and uncoated wire surfaces revealed the topography and demonstrated homogeneity with an almost equal distribution of the prepared coating layers. EDX spectroscopy analysis is used to determine the essential elemental composition and percentile of a substance (Figs. 6 and 7). This analysis confirmed the presence of Ag within the coated orthodontic arch-wire segments compared with the control groups, indicating the attachment and presence of Ag coating the specimens in the nanocomposite matrices of CS and PVA along with carbon (C), oxygen (O_2_), Ni and Ti. The presence of AgNPs was indicated by the presence of dark spots detected on the NiTi wires. The CS-Ag coated NiTi wires showed fewer surface irregularities than did the PVA-Ag coated wires. The CS-Ag coated NiTi wires exhibited a very homogeneous, dense layer surrounding the wire, ensuring that the AgNPs attached to the wire surface with a smooth, fully surrounding layer, resulting in more pigmented spots around the wire. After intentional scraping, SEM images revealed a well-defined continuous layer of nanocomposite layer with dark spots adhered to the wires. In contrast, the AgNPs and PVA-Ag coated wires had a thinner layer with lighter spots.

#### Release of Ni and Ag ions

The release of Ag and Ni ions from the coated wires was calculated after 24, 48, 96 and 192 h for each NiTi wire sample. AAS results showed that the Ag ions in all the AgNPs and nanocomposites coated samples were the highest after 24 h at a maximum of 0.18 ppm and were inversely proportional to time. The release rates ranged between 0.03 and 0.18 ppm, while the Ni ions in all the samples ranged between 0.12 and 0.33 ppm, with a continuous decrease in the release rates of both elements up to 192 h (8 days).

## Discussion

Orthodontic wires are essential tools for the correction of dental malocclusions in a process associated with bacterial accumulation and biofilm formation. Surface coatings are a simple means to augment the chemical and biological activities of dental appliances. Antibacterial and, more attractively, the antibiofilm activities are the most targeted properties, where these coatings can sustain the release of antibacterial agents for the desired period after implantation. These active coatings ensure the release of encapsulated bactericidal nanoparticles at effective concentrations that can inhibit bacterial colonization and biofilm formation. Providing the ultimate required niche for orthodontic treatment without the disadvantages and obstacles traditionally faced during orthodontic procedures [33, 34].

Coating these wires with antimicrobial agents, to decrease microbial colonization is the main aim of this study. Here, we augmented the biological activity of NiTi orthodontic wires (antimicrobial and antibiofilm) by coating them with AgNPs and Ag nanocomposites (PVA-Ag and CS-Ag). The recorded antimicrobial activity of these wires confirms the significance of the added layer in the fight against microbial attachment and colonization. These results showed the superiority of CS-Ag coated wires, as they retained AgNPs, where they acted as an encapsulated form of Ag nanocomposite with maximum rates of inhibition against Gram-positive bacteria such as *Staphylococcus aureus*, *Streptococcus mutans* and *Enterococcus faecalis*, which are the most predominant bacterial species in the oral cavity.

Similar results against Gram-positive bacteria were reported in multiple studies [2, 28, 35]. Other studies, such as Nafarrate-Valdez et al., 2022, have tested the antimicrobial activity of AgNPs and CuNPs coated stainless steel (SS) wires against *Streptococcus mutans*. Other studies have shown the ability of AgNPs coated orthodontic bands to cause significantly greater antimicrobial activity than ZnO coated bands [36, 37].

In this study, we explored the effects of nanoparticles and nanocomposite coatings on Gram-negative bacteria and demonstrated their significant microbial inhibitory effects on *Pseudomonas* spp. and *Acinetobacter* spp. We have noted that studies have not tested the antibacterial activity of NiTi coated wires against Gram-negative bacteria, under the assumption that Gram-positive are the most dominate and represent the highest rates of recorded infections. However, Gram-negative infections are present in hospitalised individuals, patients with poor oral hygiene and patients with chronic periodontal infection [38–40]. The results confirmed the ability of these coated NiTi wires to cause significant biofilm inhibition. CS-Ag coated wires showed the most meaningful results against Gram-negative biofilm-former multidrug-resistant bacteria. Our results confirmed that the attachment of AgNPs to wires can be achieved and the Ag nanocomposite (CS-Ag) inhibited bacterial attachment, growth, and colonization. In this study, we highlighted the efficacy of these coated NiTi wires against Gram-negative bacteria, as this is the current and upcoming critical category of microorganisms as stated by the WHO. This type of infection has become more prevalent after the emergence of COVID-19 and the dominance of antibiotic resistance, with a higher susceptibility of individuals to infections, especially Gram- negative infections.

This antibacterial and antibiofilm activity of AgNPs can be attributed to several factors. These factors include the following: synthesis route by using an environmentally friendly pathway, a small particle size less than 50 nm, a spherical shape form of nanoparticles with a well-defined zeta potential, high penetration power and accessibility to bacterial cell (Gram-positive and Gram-negative) that are able to inhibit bacterial growth by causing bacterial cell disturbance and death [29, 41]. In our study, the significant bacterial growth inhibition can be attributed to the small sized AgNPs, average size of 20 nm, which have acted against all tested bacterial isolates. Furthermore, AgNPs have shown more antimicrobial affinity against Gram-negative than Gram-positive bacteria. This can be explained by the difference in bacterial structure and the thin LPS layer in Gram-negative bacteria that may function as attraction sites for nanoparticles. On the other hand, Gram-positive bacteria have thick layer of peptidoglycan that act as barricades against the flow of AgNPs towards the bacterial cell [42]. The antimetabolic activity of synthesized nanoparticles and coated orthodontic wires against formed biofilms need to be assessed in future research to be able to have a better understanding to the mechanism of action of nanoparticles and coated material on the biofilm metabolic activity.

Using carriers and scaffolds for these biologically synthesized nanoparticles has enhanced the sustainability and survival of these particles, increasing the effectiveness and durability of antimicrobial and antibiofilm activity [30, 43]. This superior characters for coated NiTi wires using biologically synthesized AgNPs and Ag nanocomposite further confirm the availability of an eco-friendly, alternative against multi drug resistant bacteria and for immunocompromised patients.

A similar deduction was reached by other studies emphasizing the augmentation effect resulting from coating orthodontic wires and their ability to prevent dental plaque formation and caries during orthodontic treatments [35, 44]. An early study showed the coating effect of adding vancomycin to a thin sol-gel film, which showed antimicrobial activity against *Staphylococcus aureus* [45]. On the other hand, some studies failed to show any change in bacterial growth using wires coated with epoxy resin and polytetrafluoroethylene (PTFE), where no antimicrobial agent was added [46]. However, the addition of AgNPs in combination with PTFE and chitosan nanoparticles with polyethylene glycol (PEG) as a coating layer has achieved significant antimicrobial activity [47, 48] confirming the essential role of AgNPs as antimicrobial agents. We can hypothesize that the combination of nanoparticles with a polymer, increases the reachability of the nanoparticles and their sustainability at the targeted site, pushing the antimicrobial and antibiofilm activities a further step by augmenting their results.

In this study we demonstrated the antimicrobial and antibiofilm activity of the nanoparticles and coated wires against normal oral flora such as *Staphylococcus spp*, *Streptococcus spp* and *Enterococcus spp* and the emerging drug resistant and biofilm forming *Acinetobacter baumannii* and *Pseudomonas aeruginosa*. This study has focused on the highly alarming emerging drug resistant and opportunistic bacterial species by developing new dental appliances, with the capability to withstand the emergence of multi drug resistance bacteria and other transient bacteria from other body sites to the oral cavity, during the long course of treatment, providing orthodontic wires with antimicrobial and antibiofilm activities against various number of bacterial infections and act as a shield against the susceptibility of the dental wires to be a niche for transient or normal oral microbial inhabitant [7, 23, 49, 50].

In this study, we used a sol-gel thin film dipping process, which ensures high starting material purity and provides 3D structural homogeneity. This process can also be conducted at low temperatures and using chemicals with a low toxicity index. Furthermore, as a nanocomposite, this form of encapsulation can protect the NiTi wires from oxidation by the nanoparticles themselves [16]. Other studies used higher temperature reaching 380 ^0^C to achieve similar results, of higher resistance to microbial colonization and lower friction and coloration properties using different polymers as carrier or nanocomposite such as PTFE [47], 2-methacryloyloxyethyl phosphorylcholine [51], parylene [52], and epoxy [53].

The AFM results revealed that the AgNPs, PVA-Ag and CS-Ag coatings significantly reduced the surface roughness of the NiTi wires. When uncoated NiTi wires have high surface roughness (0.1 and 1.3 micrometres) [54, 55], nanocoating with AgNPs alone or with Ag nanocomposites decreases these values to the nanometer scale and decreases the surface roughness significantly. Similar results were obtained by previous studies using various nanomaterials as coatings [56, 57], confirming the effectiveness of coating on future improvements targeted by research to increase the surface quality, corrosion resistance and biocompatibility of NiTi wires. Here, we must indicate that surface roughness is directly proportional to the friction coefficient, where coating NiTi wires not only decreased the surface roughness but also impacts the friction of the wires. This can be explained by the ability of nanoparticles to function as a buffer or a separation layer that can minimize contact and decrease surface sharpness. Another aspect that can be discussed is the continuous release of the nanomaterial, which acts as a lubricant that has been washed and provides a slippery route, with a positive impact on the friction forces [58]. Furthermore, SEM images and EDX analysis confirmed a uniform coating layer on the NiTi wire surface of the AgNPs and Ag nanocomposites with available Ag, especially in the CS-Ag coated NiTi wires, indicating the ability of this coating material to retain Ag and consequently explain the highest antimicrobial and antibiofilm activities against all tested bacterial isolates. Similar results were presented by previous studies using either AgNPs or CS as a coating material for orthodontic wires [11, 16, 47] and similar results were recorded using nano-ZnO as a coating material [59]. Furthermore, we can speculate that coating orthodontic wires can impact their corrosion resistance and limit metal ions release. This need to be further studied, where normally formed oxide layer can be affected by orthodontic sliding mechanics and the acidic pH created by colonizing bacteria compromises this protective oxide layer and accelerates the corrosion process. Previous studies have suggest that coating materials can delay and optimistically prevent the corrosion process and limit the release of Ni ions release [11, 55, 60].

By performing an in vitro study, we acknowledge the controlled experimental conditions and results provided in the laboratory in comparison to in vivo studies; however, we pave the way for guidelines and preliminary conclusive results for the upcoming animal and clinical studies that should address the advantages of coating by metal nanoparticle encapsulation to reduce all metal-ion-related allergies and the consequential clinical effects of the coating in clinical practice and its effects on microbial growth, tooth surface demineralization, and periodontal diseases.

Finally, during the long course of orthodontic treatment, that may last for many months, these coated wires will provide antibacterial and antibiofilm capabilities with a prophylactic edge, if and when patients are exposed to antibiotic resistant non-oral bacterial species that may present as transient unwelcome guests or an opportunistic pathogens.

## Conclusion

The AgNPs and nanocomposite coated NiTi wires showed significantly greater antibacterial and antibiofilm activities, especially the CS-Ag coated wires, which exhibited the highest rates of bacterial and biofilm inhibition against both Gram-positive and Gram-negative bacteria. All three types of coatings impacted the surface roughness, topography, and release of ions, with positive implications for future in vivo studies using coated NiTi wires.

## Electronic supplementary material

Below is the link to the electronic supplementary material.

Supplementary Material 1

## Data Availability

The datasets generated during and/or analysed during the current study are available from the corresponding author upon reasonable request.

## Abbreviations

AgNPs: Silver nanoparticles
AFM: Atomic force microscopy
CLSI: Clinical and Laboratory Standards Institute
CFU: Colony forming unit
CS: Chitosan
CNx: Carbone Nitride
DLS: Dynamic light scattering
F: Fluoride
MHA: Mueller-Hinton agar
Ni: Nickel
NPs: Nanoparticles
NiTi: Nickel–titanium
OD: Optical density
PBS: Phosphate-buffered saline
PI: Polydispersity index
Ppm: Part per million
PTFE: Polytetrafluoroethylene
PEG: Poly (ethylene glycol)
SR: Surface roughness
SD: Standard deviation
SEM/EDX: Scanning electron microscope equipped with energy dispersive X-ray spectroscopy
TEM: Transmission electron microscopy
WS2: Tungsten disulfide
WSLs: White spot lesions
ZnO: Zinc oxide
