# Supplementary material for: Comprehensive evaluation of the antibacterial and antibiofilm activities of NiTi orthodontic wires coated with silver nanoparticles and nanocomposites: an in vitro study
Source: BMC Oral Health. 2024 Nov 5;24:1345. doi: 10.1186/s12903-024-05104-w (PMC11539822; doi:10.1186/s12903-024-05104-w)
Supplement: Supplementary file 1 — Supplementary Material 1 [file 12903_2024_5104_MOESM1_ESM.docx]

*I have worked on these figures to be clearer and added the last updated version in the manuscript- after revision but her I attach supplementary images.*

**Fig (I) supplementary:**

The Sem images of AgNPs coated NiTi wires at different magnification power

**(A)**


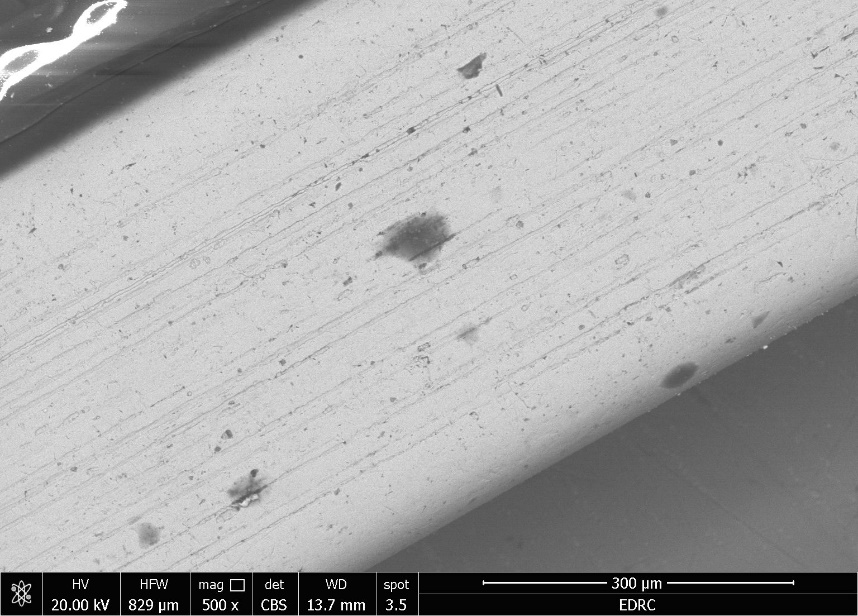

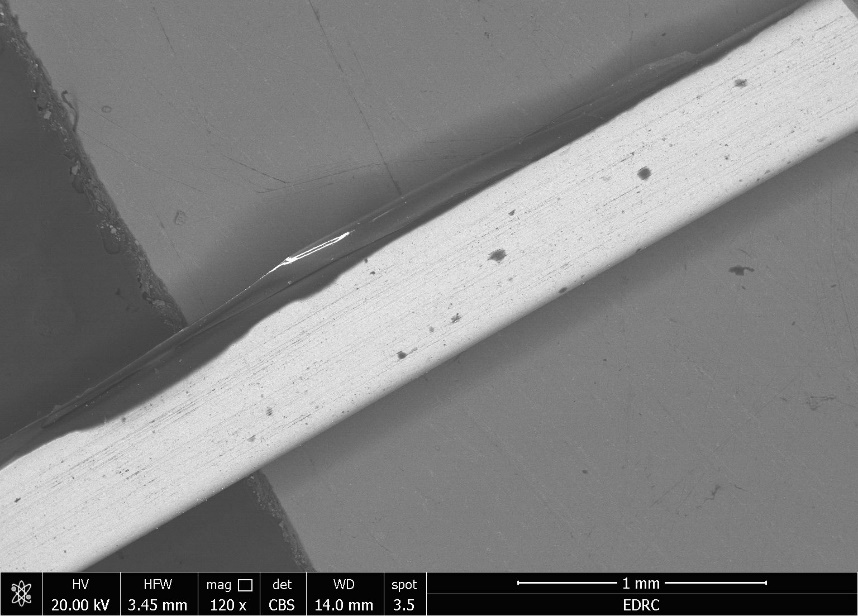


**(B)**

**(C)**

**Fig (II) supplementary:**

The Sem images of PVA-Ag coated NiTi wires at different magnification power


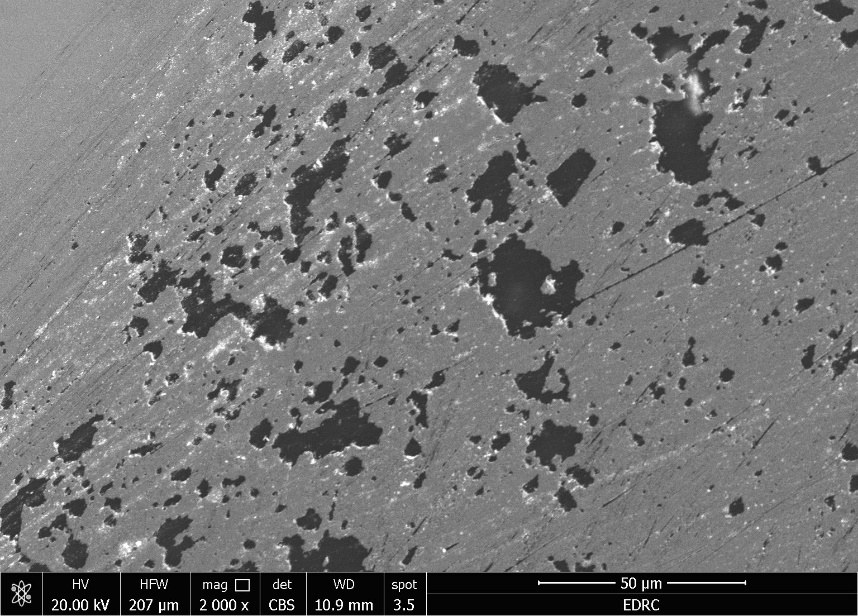

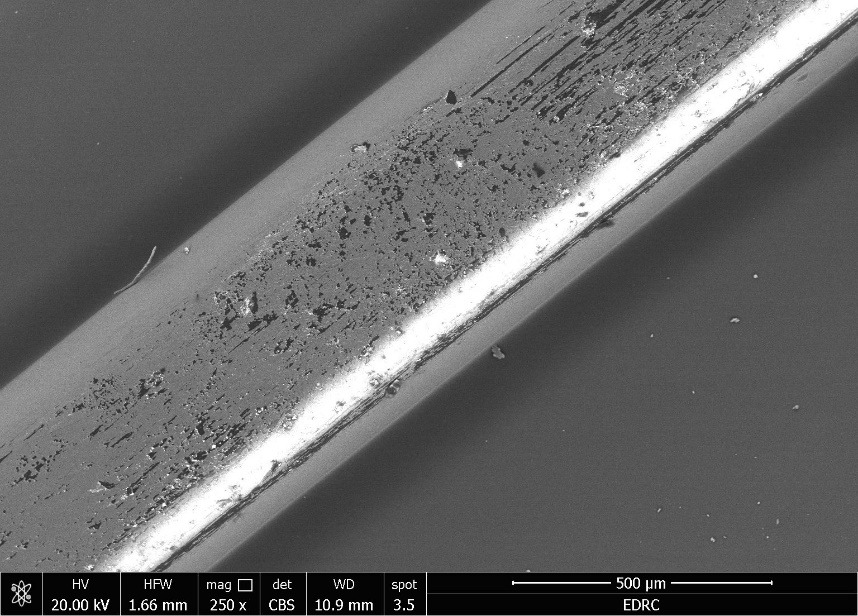


**Fig (III) supplementary:**

The Sem images of CS-Ag coated NiTi wires at different magnification power

**
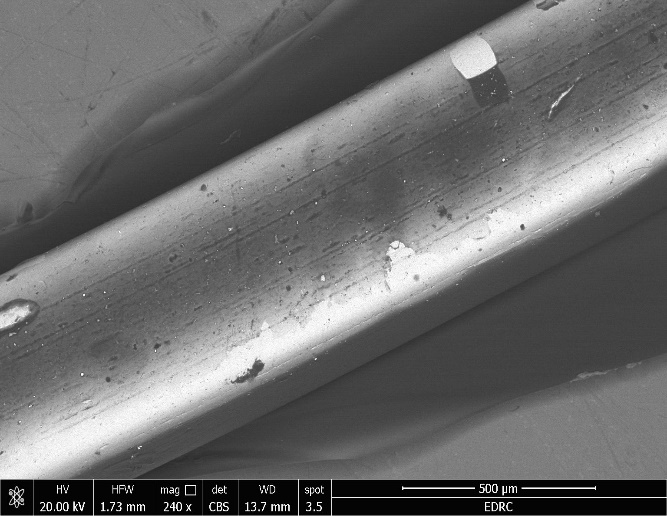

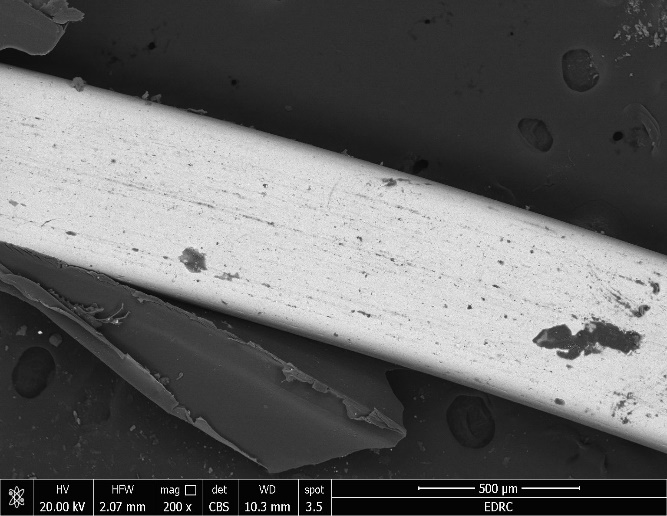

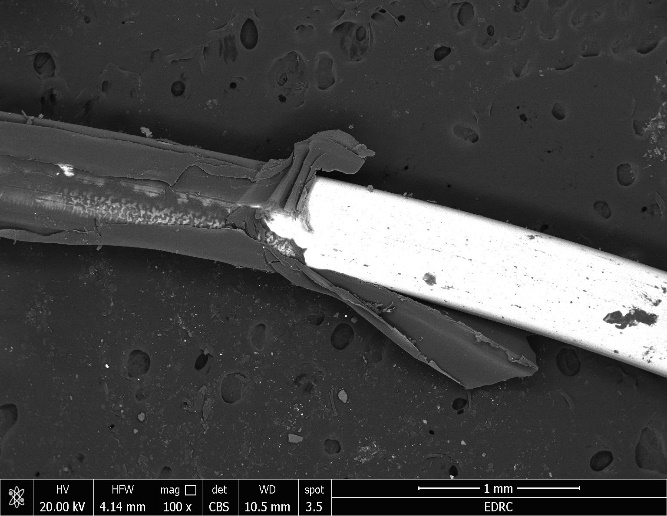
**
